# A case report of Muir-Torre syndrome in a woman with breast cancer and MSI-Low skin squamous cell carcinoma

**DOI:** 10.1186/s13053-017-0066-9

**Published:** 2017-05-12

**Authors:** Caroline Kientz, Marie-Odile Joly, Laurence Faivre, Alix Clemenson, Sophie Dalac, Côme Lepage, Caroline Chapusot, Caroline Jacquot, Renaud Schiappa, Marine Lebrun

**Affiliations:** 1 Department of Genetics, Hôpital Nord, CHU Saint Etienne, 42055 Saint-Etienne Cedex 2, France; 2 Department of Central Anatomy and Pathological Cytology, Edouard Herriot Hospital, Hospices Civils de Lyon, 69437 Lyon Cedex 3, France; 3 Claude Bernard University Lyon 1, University of Lyon, 69622 Villeurbanne Cedex, France; 4 Center of Genetics, Children’s Hospital, 14 rue Gaffarel, BP 77 908, 21079 Dijon Cedex, France; 5 Department of Pathology, Hôpital Nord, CHU Saint Etienne, 42055 Saint-Etienne Cedex 2, France; 6 Department of Dermatology, University hospital F. Mitterrand, 14 rue Gaffarel, BP 77 908, 21079 Dijon Cedex, France; 7 Inserm U 866, Hepatogastroenterology and Digestive Oncology, University Hospital F. Mitterrand, 14 rue Gaffarel, BP 77 908, 21079 Dijon Cedex, France; 8 University of Burgundy, Esplanade Erasme, 21078 Dijon, France; 9 Genetic Cancers Platform, Burgundy Area, Pathology Department, University Hospital F. Mitterrand, 2 Rue Ducoudray, BP 37013, 21070 Dijon, France; 10 Research center, Epidemiology and Bioinformatics Unit, Centre Antoine Lacassagne, 33 avenue de Valombrose, 06189 Nice cedex 2, France

**Keywords:** Muir-Torre syndrome, Lynch syndrome, MSI, MSI-L, Breast cancer, Squamous cell carcinoma, Sebaceous adenoma, MMR, MSH2

## Abstract

**Background:**

The tumor spectrum in the Lynch syndrome is well defined, comprising an increased risk of developing colonic and extracolonic malignancies. Muir-Torre syndrome is a variant with a higher risk of skin disease. Patients have been described carrying mutations in the mismatch repair genes and presenting tumors with unusual histology or affected organ not part of the Lynch syndrome spectrum. Hence, the real link between Lynch syndrome, or Muir-Torre syndrome, and these tumors remains difficult to assess.

**Case presentation:**

We present the case of a 45-year-old-woman, diagnosed with breast cancer at 39 years of age and skin squamous cell carcinoma (SCC) at 41 years of age, without personal history of colorectal cancer. The microsatellite instability analysis performed on the skin SCC showed a low-level of microsatellite instability (MSI-Low). The immunohistochemical expression analysis of the four DNA mismatch repair proteins MLH1, MSH2, MSH6 and PMS2 showed a partial loss of the expression of MSH2 and MSH6 proteins. Germline deletion was found in *MSH2* gene (c.1277-? _1661 + ?del), exon 8 to 10. Then, at 45 years of age, she presented hyperplastic polyps of the colon and a sebaceous adenoma.

**Conclusion:**

Squamous cell carcinomas have been described in Lynch syndrome and Muir-Torre syndrome in two studies and two case reports. This new case further supports a possible relationship between Lynch syndrome and squamous cell carcinoma.

## Background

Muir-Torre syndrome (MTS) was described by Muir and Torre as a rare autosomal dominant genodermatosis [1, 2] and was characterized by skin tumors such as sebaceous adenomas, sebaceomas, sebaceous carcinomas or keratoacanthomas and internal malignancies. The link between Hereditary Non-Polyposis Colorectal Cancer (HNPCC) and MTS was made in 1981 by Lynch et al. [3]. HNPCC, also known as Lynch syndrome (LS), is an autosomal dominant tumor predisposition for colorectal (CRC), endometrium, stomach, pancreas, ovarian, ureter and renal pelvis, and biliary tract cancers, caused by a germline mutation in one of the mismatch repair (MMR) genes *MLH1*, *MSH2*, *MSH6*, *PMS2*. Deletions of the 3’ end of the *EPCAM/TACSTD1* gene lead to *MSH2* promoter hypermethylation, causing LS. MTS patients are more often carriers of *MSH2* gene mutation [3].

Identification of patients affected by LS is now largely based on microsatellite instability (MSI) analysis. This is supplemented by analysis of MLH1, MSH2, MSH6 and PMS2 protein expression by immunohistochemistry (IHC). MSI is the hallmark of LS tumors and involves an accumulation of errors in the microsatellite sequences. MSI is found in tumors from patients carrying a constitutional mutation of MMR genes and in sporadic cases [4, 5].

Detection of MSI is based on markers, generally using a National Cancer Institute-recommended panel, the Bethesda Panel [4] (D2S123, D5S346, D17S250, BAT26, BAT25). A high degree of instability (MSI-High) is determined when at least two markers are unstable. When there is no instability, tumor is classified as microsatellite stable (MSS). If one marker shows instability, tumors are classified as stable (MSS) or as a low-level MSI (MSI-Low). In this case, there is no consensus [5] and the classification is laboratory-dependent. Loss of MLH1, MSH2, MSH6 or PMS2 protein expression can be detected by IHC. MMR proteins form heterodimeric complexes (MSH2/MSH6 and MLH1/PMS2). IHC analysis has the advantage of indicating the MMR gene likely to be altered [6].

## Case presentation

A 40-year-old woman, originally from the Maghreb region, had a genetic consult because at 39 years of age, she presented breast cancer, a high grade in situ ductal carcinoma, with suspected microinvasion. She underwent mastectomy, but received neither chemotherapy nor radiotherapy. She had endometriosis and resection of three hyperplastic polyps of the endometrium. She had first-degree relatives with cancers, one sibling died at 14 years of age of brain tumor and another sibling had colorectal carcinoma at 47 years of age. Her mother had surgery at 52 years of age for a right-side moderately differentiated colon adenocarcinoma, and died 1 year later. There was no cancer in the paternal branch.

Familial history was not sufficient to perform *BRCA1* and *BRCA2* gene analysis. No cancer was available for analysis by IHC or for MSI and the patient had no contact with her sibling with CRC. IHC and MSI could not be performed on breast cancer (BC) nor on endometrial hyperplasia for technical reasons.

**Fig. 1:**
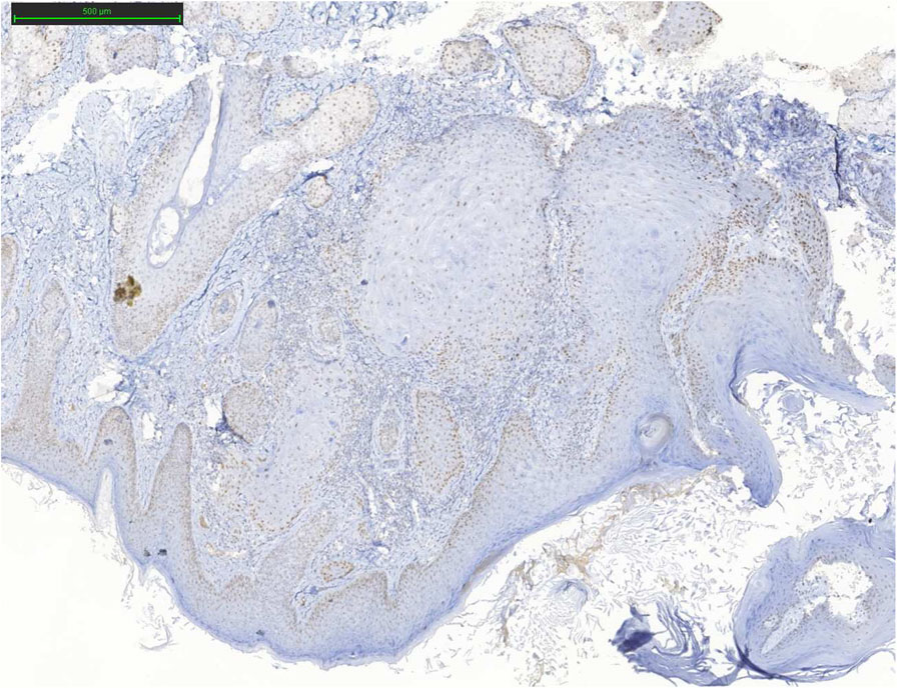
MLH1 protein expression in squamous cell carcinoma by IHC

**Fig. 2:**
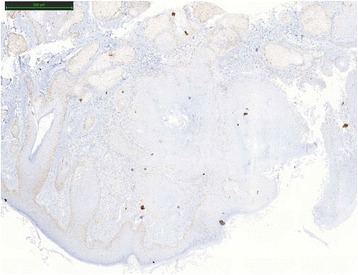
Partial loss of MSH2 protein expression in squamous cell carcinoma by IHC

**Fig. 3:**
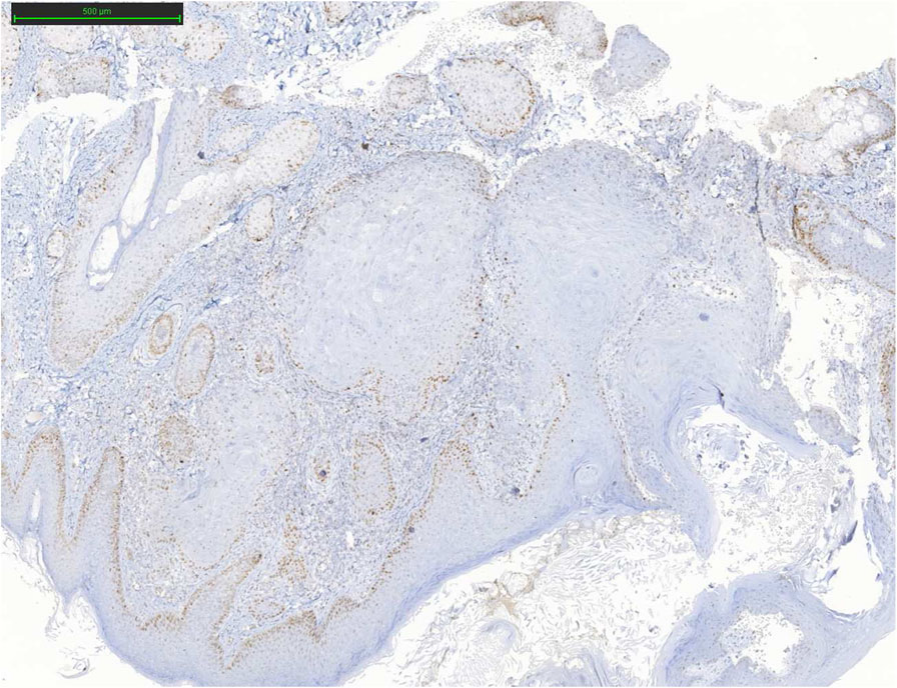
Partial loss of MSH6 protein expression in squamous cell carcinoma by IHC

**Fig. 4:**
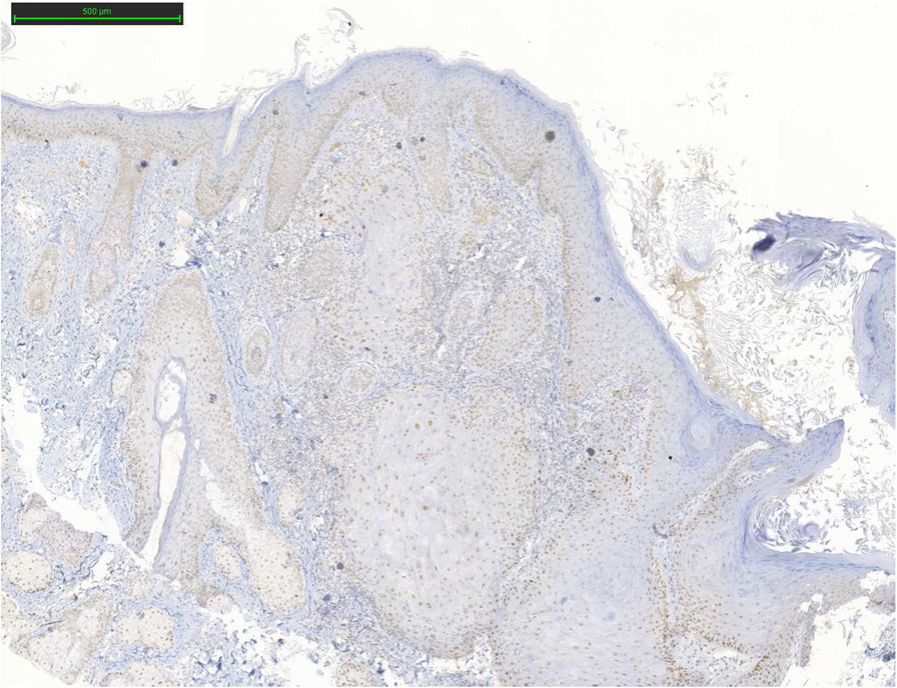
PMS2 protein expression in squamous cell carcinoma by IHC

One year after the first genetic consult, the patient presented an infiltrating, well-differentiated and keratinizing skin squamous cell carcinoma of the nose. Five markers, BAT25, BAT26, NR21, NR24 and Mono27 (Promega MSI analysis system) were used to determine the level of instability of the SCC. BAT25 marker was unstable. The results were intermediate for BAT26. Not enough healthy tissue was available and it was contaminated by tumor. SCC was classified as MSI-Low. Immunohistochemical expression analysis was made with MLH1 (clone G168/728, Ventana), MSH2 (clone G219-1129 BD Biosciences), MSH6 (clone 44 BD Biosciences) and PMS2 (clone A16-4, Pharmigen) antibodies. Expression of MSH2 and MSH6 proteins were heterogeneous and only at the periphery of the lesion (Figs. 1, 2, 3 and 4).

Hence, taking into account family history and the results of IHC and MSI, a genetic test was suggested. Direct sequencing of entire coding regions and intron/exon boundaries of *MLH1* and *MSH2* genes and last exon of *EPCAM* gene was performed. Germline deletion was found in *MSH2* gene (c.1277-? _1661 + ?del), exon 8 to 10.

At 45 years of age, the patient presented eight hyperplastic rectal polyps and two fibrous skin lesions, one in the back, corresponding to a sebaceous adenoma, the second, on the thigh, corresponding to epithelial hyperplasia. IHC and MSI analyses were performed on the sebaceous adenoma showing three unstable markers (BAT25, BAT 26 and Mono27), thus corresponding to MSI-H tumor. There was a total loss of expression of MSH2 and MSH6 protein, characteristic of MMR deficient tumors, while in the SCC loss was only in the center of the tumor.

## Discussion and conclusions

Neither breast cancer nor SCC is part of LS tumor spectrum, but both have already been described in patients with MMR gene defect. SCC was described in two studies and two case reports. In 2002, Mathiak et al. [7] studied 28 skin lesions from patients with MTS, of which one was a SCC. In 2006, Gray et al. [8] examined the prevalence of MSI in 22 confirmed invasive SCC using Bethesda Panel. Only one showed instability and was MSI-Low. In 2014, Amjad et al. [9] reported the first case of SCC in the duodenum, in an LS patient known to have *MSH2* gene deletion. In 2015, Sorscher [10] presented the case of a 54-year-old patient with SCC and *MLH1* gene mutation who presented colorectal cancer 2 years earlier.

Risk of breast cancer in Lynch syndrome is still unclear and some studies have been conducted to resolve this question. Win et al. [11] reviewed the literature published before December 2012 including 15 molecular studies analyzing MSI and IHC in breast cancer from patients with MMR gene mutation, and 21 studies which evaluated risk of BC in the LS context [11, 12]. They concluded that, in some LS patients, MMR deficiency can be linked to an increased risk for breast cancer. Harkness et al. [13] recently highlighted an increased risk of BC in female *MLH1* mutation carriers with 18.6% cumulative risk at 70 years, whereas the risk for the United Kingdom population is around 7.5–8%. *MSH2* mutation carriers have an 11.2% cumulative risk of breast cancer at 70 years. In 2013, Vasen et al. [14], a group of European experts, published revised guidelines for the clinical management of LS. They discussed the results of Barrow et al. [15] and Engel et al. [16], who reported an increased risk of BC in Lynch Syndrome after the age of 40 years, with the highest risk in *MLH1* carriers.

Based on our review of the literature, this is the first Muir-Torre syndrome case with skin squamous cell carcinoma and breast cancer at early onset, without personal history of CRC or adenomatous polyps. We have no scientific arguments to establish a link between this patient’s breast cancer and MTS but we can pinpoint the relatively early age at which she presented her tumor.

There is no consensus for MSI-Low tumor classification. In this case, if the lab which performed IHC and MSI analysis had considered MSI-Low tumor as MSS, the diagnosis of MTS could have been missed. MSI-Low tumors should be monitored carefully and should not be classified as stable too hastily. When there is doubt about the results, it is essential to perform both IHC and MSI. The results must be interpreted with caution when involving a tumor that is not part of the Lynch syndrome tumor spectrum and take family history into account. Promega MSI Panel and Bethesda panel have been proved effective for the detection of instability in colorectal and uterine tumors [5, 17]. However, they are less suitable for extra-colonic tumors. A more systematic use of additional markers in extra-colonic tumors could result in a more accurate classification of tumors classified MSI-Low.

Expression of MSH2 and MSH6 proteins was altered but there was no total loss of expression. In our opinion, it would be interesting to consider the partial expression of MMR proteins as a potential indication of an underlying mutation, even in unusual tumors that are not considered part of the Lynch syndrome spectrum.

This is a new case of a patient carrying MMR mutation with tumors not part of LS. Breast cancer and SCC are relatively common, making it difficult to establish the real link between this kind of tumor and MMR germline defects. However, as testified by the literature, there are more and more cases of breast cancer being described in Lynch syndrome patients. It may be appropriate to start taking into account this increased risk and propose earlier monitoring of LS women’s breasts.

This new case also leads us to consider the possibility that MMR gene defect may result in an increased risk of SCC, but large cohort studies with a long follow-up should be conducted.

From a technical perspective, we are convinced that performing both IHC and MSI is crucial for the interpretation of the results and that MSI-Low should not be considered as MSS. Performing both IHC and MSI may not be a cost-effective strategy, but could help identify more patients with Lynch syndrome and prevent cancer by appropriate surveillance.

## Abbreviations

BC: Breast cancer
CRC: Colorectal cancer
HNPCC: Hereditary non-polyposis colorectal cancer
IHC: Immunohistochemistry
LS: Lynch syndrome
MMR: Mismatch repair
MSI: Microsatellite instability
MSI-L: Microsatellite low instability
MSS: Microsatellite stable
MTS: Muir-Torre syndrome
SCC: Squamous cell carcinoma

## References

[1] Muir EG, Bell AJ, Barlow KA (1967). Multiple primary carcinomata of the colon, duodenum, and larynx associated with kerato-acanthomata of the face. Br J Surg.

[2] Torre D (1968). Multiple sebaceous tumors. Arch Dermatol.

[3] Arshad A, D’Angelis CA (2014). Sebaceous tumors of the skin and Muir Torre Syndrome—a mini review. N A J Med Sci.

[4] Umar A, Boland CR, Terdiman JP, Syngal S, de la Chapelle A, Rüschoff J (2004). Revised Bethesda guidelines for hereditary nonpolyposis colorectal cancer (Lynch syndrome) and microsatellite instability. J Natl Cancer Inst.

[5] De la Chapelle A, Hampel H (2010). Clinical relevance of microsatellite instability in colorectal cancer. J Clin Oncol.

[6] Boland CR, Koi M, Chang DK, Carethers JM (2008). The biochemical basis of microsatellite instability and abnormal immunohistochemistry and clinical behavior in Lynch syndrome: from bench to bedside. Fam Cancer.

[7] Mathiak MI, Rutten A, Mangold E, Fischer HP, Ruzicka T, Friedl W (2002). Loss of DNA mismatch repair proteins in skin tumors from patients with Muir-Torre syndrome and MSH-2 or MLH-1 germline mutations: establishment as a screening test. Am J Surg Pathol.

[8] Gray SE, Kay EW, Leader M, Mabruk M (2006). Enhanced detection of microsatellite instability and mismatch repair gene expression in cutaneous squamous cell carcinomas. Mol Diag Ther.

[9] Amjad AI, Singhi AD, Balaban EP, Dudley B, Brand RE, Bahary N (2014). First reported case of a squamous cell carcinoma arising in the duodenum in a patient with Lynch syndrome. Int J Clin Exp Pathol.

[10] Sorscher S (2015). A case of squamous cell carcinoma of the skin due to the molecularly confirmed Lynch Syndrome. Hered Cancer Clin Pract.

[11] Win AK, Lindor NM, Jenkins MA (2013). Risk of breast cancer in Lynch syndrome: a systematic review. Breast Cancer Res.

[12] Castellsagué E, Foulkes WD (2015). Lynch syndrome: five unanswered questions. Clin Genet.

[13] Harkness EF, Barrow E, Newton K, Green K, Clancy T, Lalloo F, Hill J, Evans DG (2015). Lynch syndrome caused by MLH1 mutations is associated with an increased risk of breast cancer: a cohort study. J Med Genet.

[14] Vasen HF, Blanco I, Aktan-Collan K, Gopie JP, Alonso A, Aretz S (2013). Revised guidelines for the clinical management of Lynch syndrome (HNPCC): recommendations by a group of European experts. Gut.

[15] Barrow E, Robinson L, Alduaij W (2009). Cumulative lifetime incidence of extracolonic cancers in Lynch syndrome: a report of 121 families with proven mutations. Clin Genet.

[16] Engel C, Loeffler M, Steinke V (2012). Risks of less common cancers in proven mutation carriers with Lynch syndrome. J Clin Oncol.

[17] Murphy KM, Zhang S, Geiger T, Hafez MJ, Bacher J, Berg KD (2006). Comparison of the microsatellite instability analysis system and the Bethesda panel for the determination of microsatellite instability in colorectal cancers. J Mol Diagn.

